# Purification of 2,3-butanediol from fermentation broth: process development and techno-economic analysis

**DOI:** 10.1186/s13068-018-1013-3

**Published:** 2018-01-25

**Authors:** Gregorius Rionugroho Harvianto, Junaid Haider, Jimin Hong, Nguyen Van Duc Long, Jae-Jin Shim, Moo Hwan Cho, Woo Kyoung Kim, Moonyong Lee

**Affiliations:** School of Chemical Engineering, Yeungnam University, Dae-dong 214-1, Gyeongsan, 38541 Republic of Korea

**Keywords:** 2,3-Butanediol purification, Commercial biofuel process, Bio-refinery process, Extraction solvent selection, Hybrid extraction-distillation, Oleyl alcohol, Techno-economic analysis

## Abstract

**Background:**

2,3-Butanediol (2,3-BDO) is a synthetic chemical compound that also can be produced by biomass fermentation, which is gaining share in the global market as an intermediate product for numerous applications, i.e. as liquid fuel or fuel additive. Several metabolic engineering fermentation strategies to enhance the production of 2,3-BDO were developed. However, the recovery of 2,3-BDO from its fermentation broth remains a challenge due to its low concentration and its solubility in water and other components. Thus, a cost-effective recovery process is required to deliver the required purity of 2,3-BDO. This paper presents a new process development and techno-economic analysis for 2,3-BDO purification from a fermentation broth.

**Results:**

Conventional distillation and hybrid extraction-distillation (HED) processes are proposed in this study with detailed optimization and economic analysis. Particularly, a systematic solvent selection method was successfully implemented to determine a good solvent for the proposed HED configuration based on numerous experimental data obtained with each solvent candidate. NRTL and UNIQUAC property methods were evaluated to obtain binary interaction parameters of 2,3-BDO through rigorous Aspen Plus regression and validated using experimental data. Total annual cost (TAC)-based optimization was performed for each proposed configuration. Even though the HED configuration required 9.5% higher capital cost than conventional distillation, placing an extraction column before the distillation column was effective in removing water from the fermentation broth and significantly improved the overall process economics.

**Conclusions:**

Oleyl alcohol was found to be the most suitable solvent for the HED of 2,3-BDO due to its high distribution coefficient and high selectivity. The proposed HED drastically reduced reboiler duty consumption and TAC by up to 54.8 and 25.8%, respectively. The proposed design is expected to be used for the commercial scale of 2,3-BDO production from fermentation process.

**Electronic supplementary material:**

The online version of this article (10.1186/s13068-018-1013-3) contains supplementary material, which is available to authorized users.

## Background

Due to the depletion of conventional crude oil resources, great attention is directed towards renewable resources. Biomass conversion produces many valuable liquid products such as bioethanol, biodiesel, 1,3-propylene glycol, and 2,3-butanediol (2,3-BDO), depending on selective enzymatic action [1]. However, process implementation for the massive production of biochemicals is not well developed due to limitations and challenges such as the development of new processes, replacement of old processes needing huge capital, low concentration of product, and recovery of product accounting for high cost due to low concentration [2].

Owing to the wide industrial application [3] as a platform and fuel biochemical [4], 2,3-BDO needs special consideration. One of the important dehydration products of 2,3-BDO—methyl ethyl ketone—is used as an industrial solvent, fuel additive, and in printing inks [5]. In addition, 2,3 BDO could also be ketalized with acetone yields acetone-2,3-BDO-ketal, which can be used as an octane booster, food additive, and in polymer formation, cosmetics, and drugs [6]. An improved process for the transformation of 2,3-BDO to acetoin is also desirable [7]. As reported earlier, the key downstream products of 2,3-BDO have a prospective global market of ~ 32 million tons per year, valued at around $43 billion [8].

An early study of the production of 2,3-BDO using microbes was performed by Harden, Norris, and Walpole [9]. *Bacillus polymyxa* was used by Ledingham and Neish on a pilot scale [10]. Conversion of lignocellulosic waste to 2,3-BDO was introduced by Flickinger [11]. To date, *Klebsiella pneumoniae*, *Serratia marcescens*, *Enterobacter aerogenes*, *Paenibacillus polymyxa,* and *Saccharomyces cerevisae* have shown potential to produce 2,3-BDO [12, 13], but the *Klebsiella* family has a higher capability than the others [14, 15]. Lee and Maddox [16] used whey permeate *K. pneumoniae* immobilized in calcium alginate to produce 2,3-BDO with the productivity of 2–3 g/L/ h^−1^. Garg and Jain [17] presented the fermentation of cellulose and hemicellulose to produce 2,3-BDO by *K. pneumoniae* for improved process economics.

A detailed study has been carried out regarding the production and route of fermentation leading to 2,3-BDO as a possible source of fuel and other valuable products [18]. However, after fermentation the broth contains a low concentration of 2,3-BDO; the remainder contains solids and soluble contents with excess water. Final output depends on raw materials and organisms used in a process. The boiling point of 2,3-BDO is about 180 °C at atmospheric pressure and it does not form an azeotrope with water. It shows easy separation from water using a conventional distillation process, although it might be not profitable due to the low concentration of 2,3-BDO in the fermentation broth.

Therefore, a key challenge is to recover the low concentration of 2,3-BDO from the fermentation broth with commercial viability. Up to now, the only promising commercial technique is the simulated moving bed (SMB), which is proposed to be implemented in a LanzaTech fermentation plant to produce anhydrous ethanol and 2,3-BDO [19, 20]. However, the drawbacks of SMB are the existence of strong non-linear behavior during design and optimization [21] and alternating loads of the pumps causing reduced lifetime and higher fault probability during operation [22]. The expensive equipment (material) used in SMB due to the need for a considerable amount of stationary phase material is also significant [23]. Therefore, several experimental techniques that are simpler and more economic than SMB such as pervaporation [24], solvent extraction [25], steam stripping [26], reactive extraction [27], and salting-out extraction [28] have been described. Although much experimental research was done at the lab scale, every technology has issues and limitations [29].

In the case of membrane separation through the pervaporation process, the activity of the membrane decreases over time as the membrane faces severe problems due to the complexity of the fermentation broth. Few studies regarding membrane separation are found in the literature. Polydimethylsiloxane (PDMS) and ZSM-5 zeolite particles in PDMS were used as membranes integrated with solvent extraction [30, 31]. A synthetic broth was used in these studies because actual fermentation broth seriously disturbs the functionality of the membrane. Membrane swelling is a major issue encountered during separation [29–31]. Reactive extraction is also an impressive methodology for 2,3-BDO; although it takes less solvent for reaction and extraction, the strongly acidic environment causes corrosion. Anti-corrosion devices need to be implemented to avoid the corrosive effect. This technique is not mature enough to be implemented on a large scale due to processing problems and has only been tested at the lab scale [27, 32].

Liquid–liquid extraction is one of the leading methodologies discussed for 2,3-BDO as a number of solvents have been used to test the extraction rate of 2,3-BDO [25]. The liquid extraction (also called solvent extraction) technique offers benefits once the recovery and recycling of solvent are made reliable to make this method economical. Several reports have explained the salt effect using the concept of solvent extraction. Salting out increases extraction efficiency and decreases the quantity of solvent needed for extraction. Selectivity and distribution coefficient are higher than liquid extraction only [28, 33–35]. In these studies, salt acts positively on extraction but it may adversely affect the distillation operation during the recovery of solvent. Recycling may have some solid particles, which can cause scaling or blockage; salt also appears in the raffinate phase, which means solvent and salt recovery will be performed separately and will account for additional cost [36]. Therefore, the reliable option for commercializing solvent extraction is to use a solvent (without salt addition) with high selectivity and distribution coefficient for 2,3-BDO.

Numerous solvents have been tested to ensure separation capability. Selection of appropriate solvent is a major goal in any solvent extraction process. The solvent selection has been done in this work and will be discussed in further sections. However, solvent extraction or any process mentioned earlier alone is not able to approach the required product purity. Hence, a process needs to be repeated two or three times or integrated with other methods to fulfill the requirements of separation [37]. A combination of solvent extraction with distillation, usually called hybrid extraction-distillation (HED), is expected to fully ensure the separation of 2,3-BDO with recovery and recycling of solvent. In solvent extraction, 2,3-BDO is extracted in an extractor by forming the organic phase, which is then separated from the water-rich phase. The organic phase is then inserted into a distillation column where the solvent is recycled back to the extractor and 2,3-BDO is recovered from the top. HED has been effectively proven and widely used for the separation of liquid mixtures [38], particularly for the recovery of products such as butanol [39], bioethanol [40], furfural [41], pyridine [42], acetic acid [43, 44], *n*-propanol [45], and ester [43].

Evaluation of 2,3-BDO purification or recovery through process design study is necessary for the commercialization of 2,3-BDO from biomass fermentation. For this reason, this work aims to propose a comprehensive process development for 2,3-BDO purification from a real fermentation broth, defining the separation with reliable analysis, simulation, optimization, and cost assessment. Herein, we focused on HED configuration and further compared with a conventional distillation process. This design will enable a clearer understanding of the thermodynamic correlations and the evaluation, solvent selection method, process simulation and optimization, and economic evaluation of the conventional distillation design and the proposed hybrid extraction-distillation process.

## Methods

**Fig. 1:**
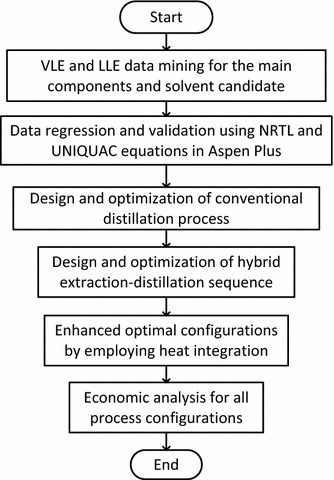
Design approach used in this study

Figure 1 provides an outline of the design approach used in this study to accomplish the objective. Binary parameters of the thermodynamic model candidates were first obtained for the main components, i.e. 2,3-BDO and water through rigorous regression and validation steps. Considering the selected thermodynamic model, several process configurations were observed using a rigorous process simulator (i.e. Aspen Plus). A cost-effective hybrid extraction distillation was proposed to purify 2,3-BDO from the fermentation broth.

### Thermodynamic model

Aspen Plus^®^V9 was used to simulate the process configurations considered. In this study, 2,3-BDO and water vapor–liquid-equilibrium (VLE) binary parameters were reproduced by employing open literature VLE data in the Aspen Plus regression function. Liquid–liquid equilibrium (LLE) data for ternary 2,3-BDO + water + selected solvent systems were also used in this study to obtain LLE binary parameters for the extractor unit in the hybrid extraction distillation configuration. The Aspen Plus physical parameter regression system provides a powerful tool for regressing and correlating experimental data to obtain binary coefficient values. The universal quasi-chemical (UNIQUAC) [46] and non-random two liquid (NRTL) [47] equations were evaluated as thermodynamic models for VLE and LLE behaviors. The adjustable parameters of the NRTL and UNIQUAC equation are described in Additional file 1. The binary parameters in the NRTL and UNIQUAC models were obtained by the generalized least-squares method to minimize the following maximum likelihood objective function (Q) [48, 49]:

1 $$Q = \mathop \sum \limits_{n = 1}^{\text{NDG}} w_{n} \mathop \sum \limits_{i = 1}^{\text{NP}} \left[ {\begin{array}{*{20}c} {\left( {\frac{{T_{e,i} - T_{m,i} }}{{\sigma_{T,i} }}} \right)^{2} + \left( {\frac{{P_{e,i} - P_{m,i} }}{{\sigma_{P,i} }}} \right)^{2} } \\ { + \mathop \sum \limits_{j = 1}^{{{\text{NC}} - 1}} \left( {\frac{{x_{e,i,j} - x_{m,i,j} }}{{\sigma_{x,i,j} }}} \right)^{2} } \\ { + \mathop \sum \limits_{j = 1}^{{{\text{NC}} - 1}} \left( {\frac{{y_{e,i,j} - y_{m,i,j} }}{{\sigma_{y,i,j} }}} \right)^{2} } \\ \end{array} } \right]$$

where NDG, NP, and NC are the number of data groups in the regression case, the number of points in data group* n*, and the number of components present in the data group, respectively. *w*_*n*_ is the weight of data group *n*; *e* is the estimated value and *m* is the measured value; *i* is the data for data point *i*; *j* is the fraction data for component *j*; and *σ* is the standard deviation of the indicated data. *T, P, x,* and *y* are the temperature, pressure, and liquid and vapor mole fractions, respectively.

To evaluate the accuracy of each thermodynamic model with the experimental data, the average absolute deviation (AAD) values were calculated for each binary pair to quantitatively validate the regression result.

2 $${\text{AAD}} = \mathop \sum \limits_{i = 1}^{n} \left( {\frac{{\left| {x_{i}^{\text{cal}} - x_{i}^{\exp } } \right|}}{n}} \right)$$

where* n* is the number of data points. $x_{i}^{\text{cal}} \,{\text{and}}\,x_{i}^{ \exp }$ refer to the calculated mole fraction and experimental mole fraction of component *i*, respectively.

### Process simulation

**Fig. 2:**
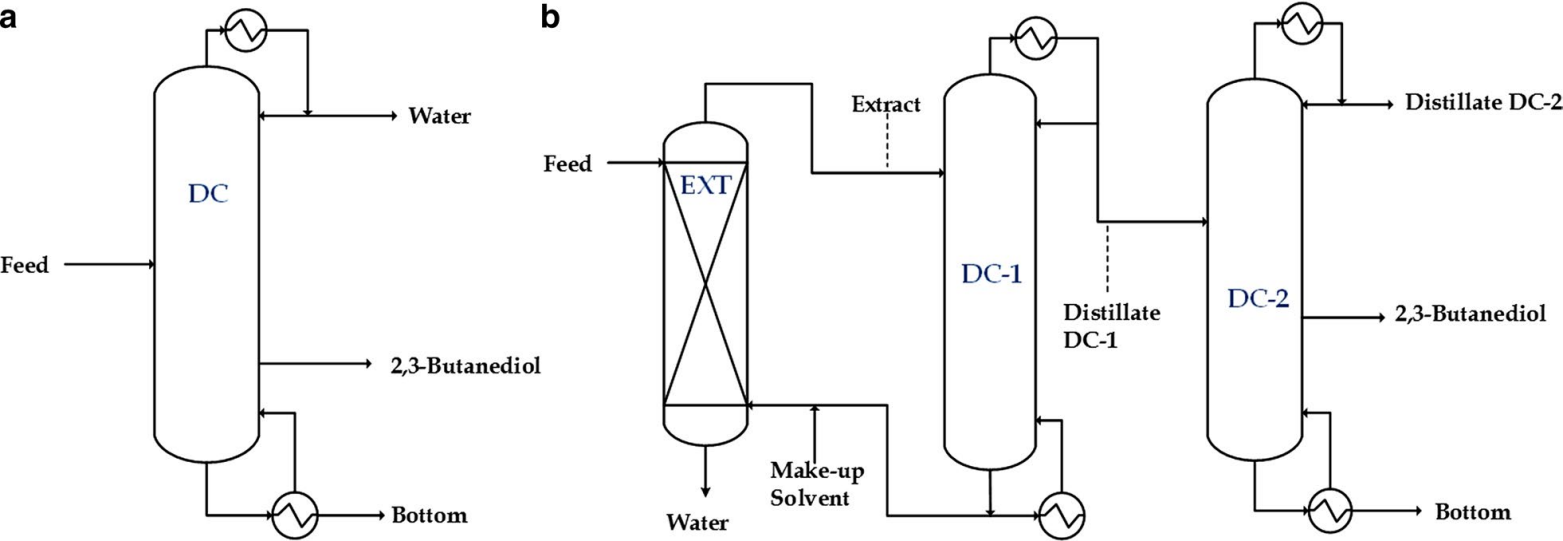
Proposed configurations for purification of 2,3-butanediol: **a** conventional distillation (base case); and **b** hybrid extraction-distillation configuration

The selected thermodynamic model was used to evaluate the proposed design for 2,3-BDO purification. Available Aspen Plus binary parameters were used for binary components of other constituents in the system. The UNIFAC group contribution method of estimation was used for the missing binary parameters. The feasibilities of process candidates for 2,3-BDO purification (Fig. 2), were examined by rigorous simulation and optimization in Aspen Plus. The feed composition and temperature were considered for the real fermentation broth, as in the previous study [50]. Table 1 lists the feed, operating conditions, and product requirements, which were based on the requirements of our industrial partner. In addition, the pressure drop for each stage in the distillation columns was set to 0.006 atm. With all proposed process configurations, the design specification function in Aspen Plus was used to achieve the qualities of product streams in the distillation column (99 wt% 2,3-BDO) by manipulating the reflux ratio. This function was also used to eliminate water in the distillate stream of the conventional distillation column (DC) and purification column (DC-2) using the boil-up ratio as the manipulating variable. The solvent recycle flow rate of the hybrid extraction-distillation configuration was calculated with the requirement of 90 wt% recovery of 2,3-BDO in the product stream and could be performed using the flowsheet design specification function in Aspen Plus.

**Table 1:** Feed mixture conditions and product requirements

Variable	Value
Feed flow rate	20,000 kg/h
Feed component and composition (wt%)	Water (87.5); 2,3-BDO (9.3); formic acid (0.027); acetic acid (0.89); lactic acid (0.0712); succinic acid (0.2026); ethanol (1.05); acetoin (0.934)
Feed and column pressure	1 atm
Feed and extractor temperature	25 °C
Product requirement	1691.28 kg/h of 2,3-BDO ≥ 99 wt%

### Sensitivity analysis and process optimization

**Fig. 3:**
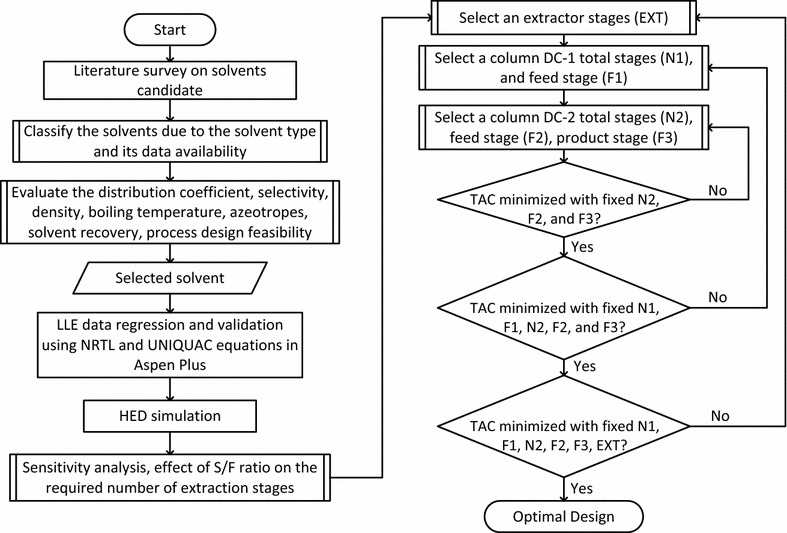
Procedure for solvent selection and its hybrid extraction distillation optimization through a sequential iterative optimization approach

Sensitivity analysis was conducted on the extraction process to identify the effect of a number of extraction stages to the mass recovery of 2,3-BDO and the required solvent flow rate. All configurations studied in this work were optimized with the help of a process optimization tool based on a sequential quadratic programming (SQP) algorithm [49, 51] in Aspen Plus. The SQP method is an efficient method for solving nonlinearly constrained optimization problems and it has been successfully employed in several previous studies [43, 52–56]. According to the sensitivity analysis which will be discussed later, the solvent rate is one of the most important variables in distillation column design and operation. In this work, the solvent rate was chosen as a manipulating variable to fulfill the output target that mentioned earlier in the previous section (product purity and recovery). The objective function is the minimum total annual cost (TAC) of the proposed configuration. The TAC, which involves the cost of the total energy and make-up solvent requirements (operating cost) and equipment purchase (capital cost), was used to evaluate the economic performances of the proposed configurations. The detailed calculation method is provided in the additional file 1. A sequential iterative optimization approach was used on both configurations in this work. For conventional distillation, TAC was minimized by increasing the number of stages until the optimal number of stages was obtained. Once the number of stages of a distillation column was changed, the feed location and side stream location were also adjusted to obtain the minimum reboiler duty. Moreover, hybrid extraction-distillation has more variables to optimize. Figure 3 shows the sequential iterative optimization procedure used for HED optimization together with the solvent selection method.

### Procedure for solvent selection

Selection of appropriate solvent is important because the solvent plays a central role in the separation process; a solvent with high extraction power has high feasibility. The main criteria for screening solvents are the distribution coefficient (*K*_*D*_) and selectivity (*α*) along with essential solvent characteristics such as density, volatility, viscosity, flammability, and polarity.

The distribution coefficient and selectivity are defined as

3 $$K_{D1} = \frac{{x_{1}^{o} }}{{x_{1}^{a} }};\,K_{D2} = \frac{{x_{2}^{o} }}{{x_{2}^{a} }};\,\alpha = \frac{{K_{D1} }}{{K_{D2} }}$$

where *K*_*D*1_ and *K*_*D*2_ are the distribution coefficients of 2,3-BDO and water, respectively, *x*_1_^*a*^ and *x*_2_^*a*^ are the mole fractions of 2,3-BDO and water in the aqueous phase, respectively, *x*_1_^*o*^ and *x*_2_^*o*^ are the mole fractions of 2,3-BDO and water in the organic phase, respectively, and *α* is the selectivity of 2,3-BDO from water.

The solvent should be cheap, easily available, immiscible with an aqueous phase, environmentally friendly, non-toxic, biologically compatible, easily recoverable from the organic phase, and have low viscosity, high *K*_*D*_, and high *α*, [57]. All the solvent candidates are described based on their experimented temperatures, partition coefficients, densities, selectivities, product separation from solvent, and ease of solvent recovery. Comparison of these parameters provides the most suitable solvent among those selected from experimental data [58].

Figure 3 describes a detailed procedure of solvent selection. In general, a systematic approach to selecting the suitable solvent for a certain component is to first determine the solvent type and the availability of data. It is the scope of this work to focus on the available sufficient LLE data and to avoid the use of a mixed solvent due to the reason mentioned in “Background”. Further, the physical properties of the target component such as density, boiling point, azeotrope formation with any component present in the mixture, distribution coefficient, and selectivity determine the next step, which includes a thermal operating range. This range will decide the stability of the product and range of boiling point of a solvent. Thermal range determines the recovery of the product either downstream or upstream depending on the boiling temperature of the solvent being used. A further step is to select suitable solvents already studied or presented in the literature with high *K*_*D*_ and *α*. Comparison based on mole fraction is necessary to calculate the *K*_*D*_ and *α* to evaluate all available solvents in the literature. The immiscibility area is usually used to check the capability or potential of solvent for the extraction process. Evaluation of shortlisted solvents needs special attention because the final design should consider the solvent having a higher capacity to extract solute and then being easily recoverable. Once solvent screening was performed using the literature, the Aspen Plus regression tool was used to obtain the binary parameters.

## Results and discussion

### Selection of solvent

To enable simulations, LLE data is necessary for process design. Thus, considering sufficient LLE data of 2,3-BDO + water + solvent, several solvents were tested earlier. The comprehensive summary of LLE data for the extraction of 2,3-BDO is shown in Table 2. Considering the advantages of a single solvent and the drawbacks of salt raised in “Background”, this work only considers single solvents for extracting 2,3-BDO from the fermentation broth. Thus, the LLE data of mixed solvents with salt [39, 40] or other components [42, 65] were excluded. Table 2 compares several solvents that have LLE data at approximately 25 °C (the real fermentation broth temperature that will be used in the design).

**Table 2:** Summary of selection criteria for available LLE data for 2,3-butanediol + water + solvent

Solvent	*T*_expt_ (°C)	K_D_ H_2_O	K_D_ 2,3-BDO	Selectivity (*α*)	Density (g/mL)	Boiling Temp. (°C)	Refs.
Ethyl acetate	25	0.17–0.28	0.22–0.63	1.3–2.8	0.897	77.1	[59]
Butyl acetate	25	0.13–0.27	0.56–0.62	2.31–4.26	0.882	126	[60]
1-Butanol	25	0.55–0.82	1.23–1.62	1.51–2.95	0.81	117.7	[61]
Isobutanol	25	0.51–0.66	1.45–1.55	2.27–2.92	0.802	108	[62]
2-Ethyl-1-hexanol	27	0.08–0.52	1.26–1.81	2.41–20.73	0.831	184.6	[63]
Oleyl alcohol	27	0.01–0.37	2.66–6.52	7.24–421.9	0.855	348.02	[64]

**Fig. 4:**
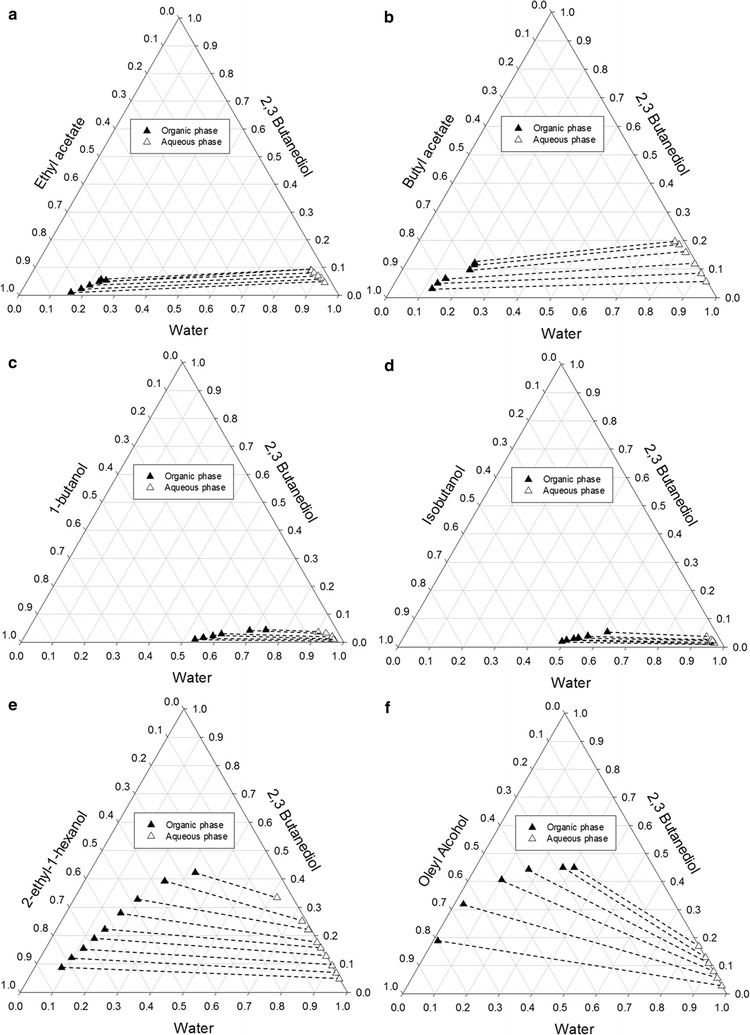
Experimental LLE tie-line data for the water + 2,3-butanediol + solvent systems: **a** ethyl acetate, **b** butyl acetate, **c** 1-butanol, **d** isobutanol, **e** 2-ethyl-1-hexanol, **f** oleyl alcohol

The density differences between solvents and the fermentation broth (1 g/mL) are significant. Further, on the basis of selectivity, all solvents show potential but several solvents each have a certain amount in the raffinate phase, as can be seen in high K_D_ H_2_O, which limits their large-scale application. According to Chen et al. [42], high distribution coefficient means the solvent requires a lower flow rate for separation and a smaller column diameter. A liquid–liquid envelope with tie lines provides details of the distribution coefficient of the solvent and whether it is good or bad for extraction. Figure 4 shows the separation behavior in terms of liquid–liquid tie lines. A solvent with *K*_*D*_ < 1 has tie lines tilted towards the solvent-rich corner while a good solvent has *K*_*D*_ > 1 with the tie lines converging towards the aqueous-rich corner in the ternary diagram [42]. As seen in Fig. 4, the *K*_*D*_ of ethyl acetate (Fig. 4a) and butyl acetate (Fig. 4b) are less than 1 and much smaller than those of the others. Ethyl acetate and butyl acetate are not suitable since their tie-lines are unfavorably tilted (low distribution coefficient), although they have sufficient selectivities. Thus, these two solvents can be eliminated at this step. As seen from Fig. 4, all alcohol solvent candidates have good abilities to separate 2,3-BDO as they have liquid–liquid tie lines tilted in the right direction from the upper-left to lower-right, which showed that higher 2,3-BDO can be obtained in the organic phase. However, 1-butanol, isobutanol, and 2-ethyl-1-hexanol form azeotropes with water, which increases the difficulty of recovering solvent from water in a subsequent distillation column. During the recovery process, this will bring water in the solvent recycle stream to the extractor along with other components. In fact, the use of either 1-butanol or isobutanol as an extraction solvent can be screened out in this work due to their relatively small LLE envelopes, although their tie-lines are favorably tilted, as shown in Fig. 4c, d.

The aforementioned solvents have limitations that retard their implementation in the extraction process. Figure 4 indicates that oleyl alcohol and 2-ethyl-1-hexanol have leading capabilities to extract 2,3-BDO from water due to their high immiscibility areas. The concentrations of these organic solvents that remain in the aqueous phase are low. However, 2-ethyl-1-hexanol has a similar boiling temperature (184.6 °C) to 2,3-BDO (180.7 °C), which eventually causes difficulties in solvent recovery. At this step, oleyl alcohol then appears as the best option for the extraction process. The characteristics of oleyl alcohol are: (1) higher distribution coefficient than other solvents, (2) highly selective for 2,3-BDO, (3) very low aqueous solubility, and (4) density difference is enough to separate. The other advantages of using oleyl alcohol as solvent are that it [66, 67]: (1) is non-toxic, (2) has small affection to forming emulsions, (3) is thermally and chemically stable, (4) is non-hazardous, (5) is easily available in excess, and (6) has large interfacial tension. The high price of oleyl alcohol and its high viscosity are the main drawbacks of this solvent [68–70].

In addition, it was found that 2,3-BDO is perfectly soluble in oleyl alcohol during the extractive fermentation process or in situ recovery process [71, 72]. As mentioned earlier, oleyl alcohol provides a compromise between high partition coefficient and lack of toxicity. For this reason, oleyl alcohol has been used more frequently than other organic solvents for acetone-butanol-ethanol extractive fermentations [73]. Oleyl alcohol has already been applied to the recovery of desired products from fermentation broths such as the recovery of butanol from a fermenter [74] and the extraction of ethanol [45]. Qureshi and Maddox [26] also reported that oleyl alcohol is a powerful candidate for the extraction of butanol.

### Thermodynamic evaluation

#### Vapor–liquid equilibrium of water and 2,3-BDO

**Fig. 5:**
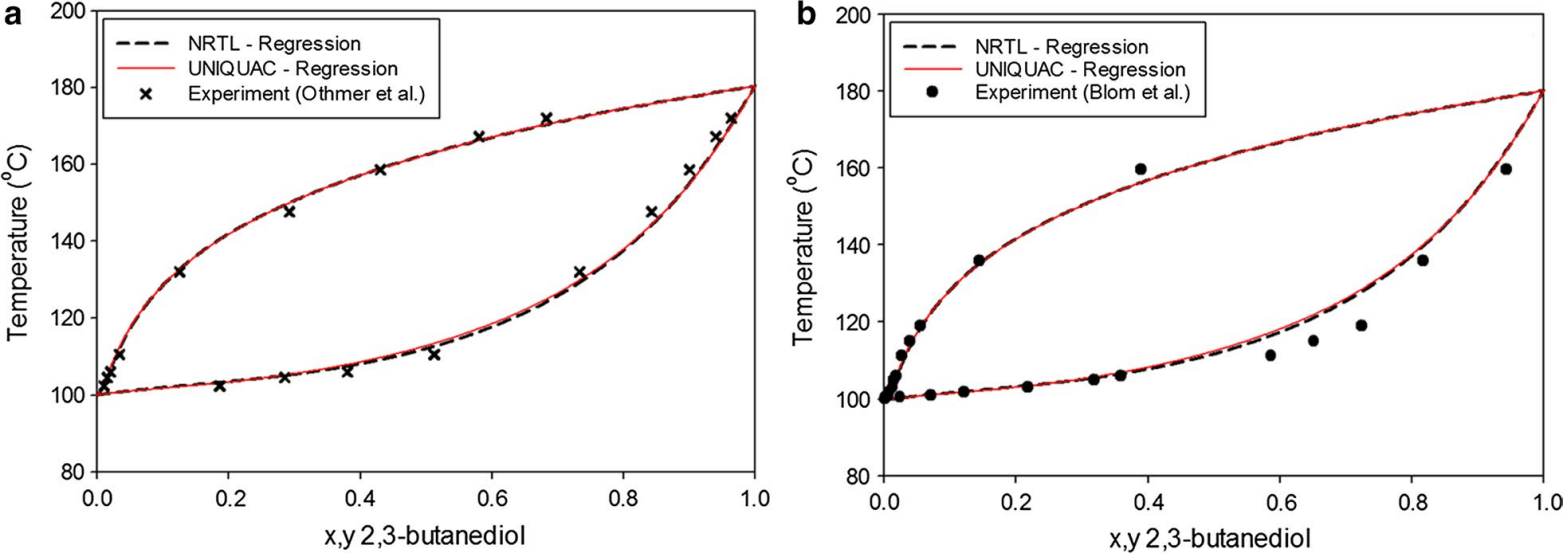
2,3-butanediol + water VLE phase diagram compared with literature values: **a** at 1 atm, **b** at 1 bar

Vapor–liquid equilibrium (VLE) experiments were performed earlier on the main constituents in the fermentation broth: 2,3-BDO and water. Blom et al. [75] examined the VLE behaviors of 2,3-BDO and water at 1 bar. Othmer et al. [76] studied the VLE of 2,3-BDO + water mixtures at 200, 350, 500, and 760 mmHg. In this study, experimental data for the VLE of the 2,3-BDO + water system were correlated using the NRTL and UNIQUAC models for process design purposes. Table 3 lists the VLE binary parameters of 2,3-BDO and water for the NRTL and UNIQUAC thermodynamic models. The binary VLE was validated using literature data [75, 76] for the T-xy equilibrium phase diagrams, as shown in Fig. 5. As shown in the figure, both NRTL and UNIQUAC matched the experimental data closely, mainly at the low concentration of 2,3-BDO that is typically obtained from the fermentation broth. NRTL matches the experimental result more closely than the UNIQUAC result, as seen from its AAD value (Table 4 and Fig. 5). Therefore, the NRTL model was chosen for simulation of the distillation column in this study.

**Table 3:** VLE binary interaction parameters of the NRTL and UNIQUAC models for 2,3-butanediol (*i*) and water (*j*)

Parameter	Thermodynamic model	
	NRTL	UNIQUAC
*a*_*ij*_	2.43182	0.623312
*a*_*ji*_	− 2.00197	0.0692492
*b*_*ij*_ (*K*)	35.5157	− 123.719
*b*_*ji*_ (*K*)	409.421	− 183.929
*c*_*ij*_	0.3	–
AAD	0.0047	0.0049

**Table 4:** LLE binary interaction parameters of the NRTL and UNIQUAC models for 2,3-butanediol (2,3-BDO), water, and oleyl alcohol (OA)

System	NRTL					UNIQUAC			
	*a*_*ij*_	*a*_*ji*_	*b*_*ij*_ (*K*)	*b*_*ji*_ (*K*)	*c*_*ij*_	*a*_*ij*_	*a*_*ji*_	*b*_*ij*_ (*K*)	*b*_*ji*_ (*K*)
2,3-BDO/Water	− 0.5863	0.567324	− 107.067	899.029	0.2	7.06203	− 34.0042	− 2388.66	10,000
2,3-BDO/OA	− 31.1741	20.1625	9998.51	− 6329.68	0.2	− 1.16437	− 17.0389	2.92684	4985.16
Water-OA	− 11.9258	− 28.0351	5808.59	9999.42	0.2	0.967516	− 22.3601	− 225.248	− 7369.56
AAD	0.0186					0.0211			

#### Liquid–liquid equilibrium of water, 2,3-BDO, and oleyl alcohol

As discussed in “Thermodynamic model”, the NRTL model has non-randomness parameters, which usually need to be fixed during data regression. As per recommendation by Khayati et al. [64], $\alpha_{ij} = 0.2$ was chosen as the value that led to the lowest residual values. Since the *d*_*ij*_ value was 0, *c*_*ij*_ is equal to $\alpha_{ij}$ in this data regression, and this value was used prior to regression. The *r* and *q* values of each component are necessary for the UNIQUAC model, as shown in Eqs. 7–13 in Additional file 1. However, the *r* and *q* values of oleyl alcohol are not available in the Aspen Plus databank. Therefore, this study used Aspen Plus to estimate the *r* and *q* values of oleyl alcohol, given its structure. The *r* and *q* values of oleyl alcohol were obtained as 12.6632 and 10.396, respectively.

**Fig. 6:**
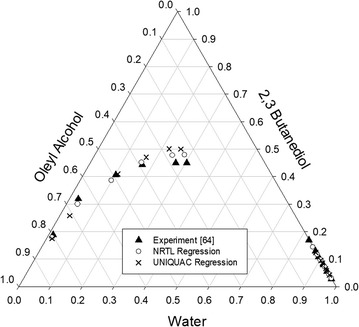
Water + 2,3-butanediol + oleyl alcohol LLE phase diagram compared with literature values at 27 °C

Liquid-liquid equilibrium data from the literature at different temperatures [64] were used for data regression. Table 4 compares the regressed binary interaction parameters of LLE and AAD values for both NRTL and UNIQUAC models. Figure 6 shows the LLE ternary diagram for both models in comparison with experimental data at 27 °C. A low AAD value indicates that both models closely correlate the experimental tie-line data and exhibit acceptable agreement. However, the correlation of the NRTL model was slightly superior to that of the UNIQUAC model. This might be due to the *r* and *q* values predicted by Aspen Plus for oleyl alcohol. Therefore, the NRTL thermodynamic model was employed to determine the liquid–liquid behavior of the mixture in the extraction column.

#### Design and optimization of conventional distillation

Distillation is a well-known separation process in the industry. Thus, this work first proposed conventional distillation as a base process configuration to purify 2,3-BDO from its fermentation broth. Based on the relative volatilities of feed component mixtures, 2,3-BDO was obtained in the side stream as the product. Meanwhile, water was completely removed from the distillate along with the other constituents. The remaining 2,3-BDO was withdrawn from the bottom stream together with the heavy components. In the proposed conventional distillation configuration, a large amount of energy is expected to be required in the reboiler since the feed from the fermentation broth is in a subcooled condition (25 °C). A high outlet stream temperature in this configuration can be used as an energy (heat) source to reduce the energy required in the reboiler by placing a heat exchanger as a feed pre-heater. The utilization of three product streams could provide significant energy savings compared to conventional distillation without heat integration. The minimum approach temperature (hot outlet–cold inlet) of each heat exchanger was designed to be 10 °C.

**Fig. 7:**
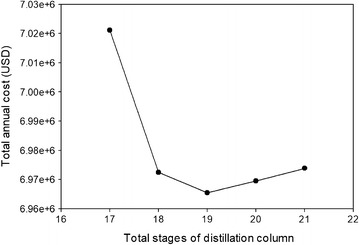
TAC plot of the conventional distillation configuration

Figure 7 shows TAC plots of the conventional distillation process at the different total stages. In each simulation run, the 2,3-BDO product specification was achieved by varying the reflux ratio of the distillation column. Once the number of stages was increased, the feed location and side stream location were also adjusted to minimize reboiler duty. The optimal feed and side stream locations were found based on the maximum allowable temperature (244 °C) in the bottom stream of DC to fulfill the constraints of using a steam reboiler and satisfying the internal flow hydraulics during hydraulic evaluation in the Aspen Plus sizing and rating function.

**Fig. 8:**
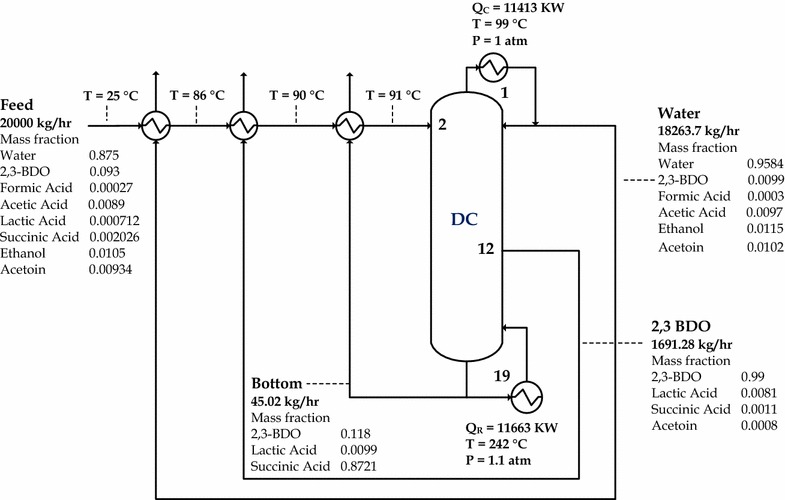
Optimal design of conventional distillation process

It was found that the optimal total stages, feed stage, and product stage are 19, 2, and 17, respectively. The optimal configuration of conventional distillation can be seen in Fig. 8. As depicted in Fig. 8, the energy consumption of the optimal conventional distillation structure can be reduced from 13,139 to 11,663 kW by utilizing product streams for preheating. The high energy consumption in the distillation process is mainly due to the excessive amount of water in the feed, i.e. 87.5 wt%. With higher volatility than 2,3-BDO, all the water should be vaporized in the distillation column and taken as the distillate product. Moreover, a fermentation broth contains other dissolved components that retard and bind the 2,3-BDO from vaporizing. Therefore, before applying a distillation process, 2,3-BDO should be pre-concentrated and other components must be removed from the broth. With the future high demand of 2,3-BDO, a cost-effective technique should be considered for purifying 2,3-BDO from its fermentation broth.

#### Design and optimization of hybrid extraction-distillation

Hybrid extraction-distillation appears as the prospective technique to be developed due to the necessity for the commercial production of 2,3-BDO. As shown earlier in Fig. 2, the main unit in this proposed design is the extraction column. The fermentation broth and solvent are, respectively, fed from the top and bottom because the solvent has a lower density than the fermentation broth. Thus, the raffinate phase contains most of the water with other constituents whereas the extract phase includes most of the solvent, 2,3-BDO, and traces of other constituents (depending on the solubility of each component in the solvent). As discussed earlier, oleyl alcohol is expected to be a good solvent with its high distribution coefficient and selectivity to separate 2,3-BDO with most of the water and to minimize the loss of solvent in the raffinate stream.

**Fig. 9:**
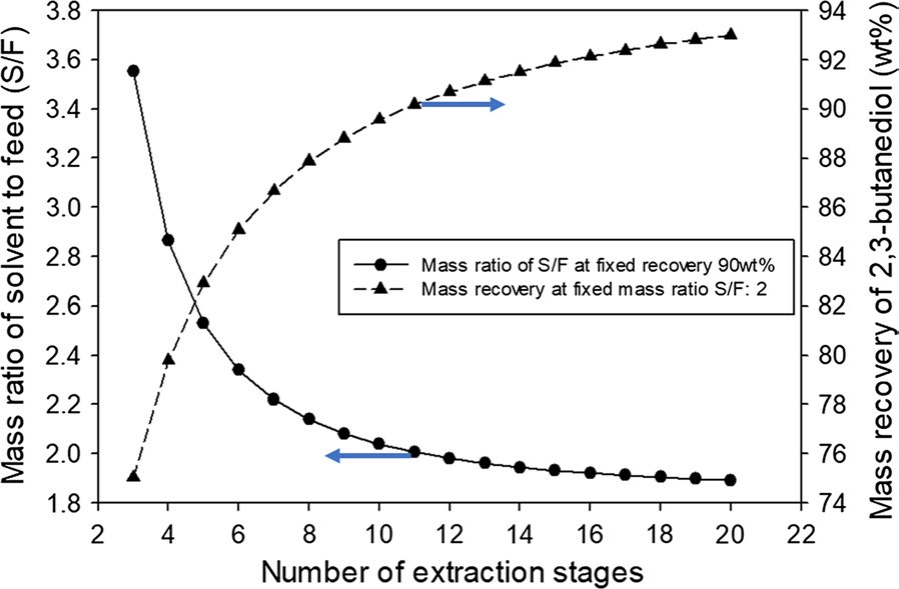
Effect of number of extraction stages on the mass ratio of solvent to feed, and mass recovery of 2,3-BDO from the extraction process

Since the ratio of extraction solvent to the feed flow rate (*S*/*F*) is an essential design variable in the HED process, its effect on the HED process was evaluated in several previous works [42, 43, 45]. As shown in Fig. 9, higher mass recovery can be obtained at fixed mass ratio *S*/*F* once the number of extraction stages is increased. Therefore, as can be seen in Fig. 9, a small number of extraction stages requires higher S/F at fixed recovery. However, higher *S*/*F* results in larger energy requirement in the subsequent distillation column since the solvent must be recycled from the recovery column (DC-1). Because the overall energy efficiency is totally based on the sum of the reboiler duties of subsequent columns, the extraction stages should be optimized along with other variables in the distillation process through the proposed optimization procedure.

Similar to the conventional distillation configuration, the extract stream from the extractor remains at low temperature (27 °C). Therefore, utilization of the outlet stream of the distillation column became advantageous as the heat source for feed pre-heating. Moreover, this heat integration provides additional benefits in the hybrid extraction distillation configuration. In the HED configuration, since the boiling point of oleyl alcohol solvent is significantly higher than that of 2,3-BDO, the solvent will be taken as the bottom stream of DC-1 and sent back to the extraction column. Therefore, the bottom temperature of DC-1 is significantly high due to the high boiling point of oleyl alcohol. In addition, it is necessary to cool the bottom stream of DC-1 prior to sending it back to the extraction column as recycled solvent. By considering this as a heat source for feed pre-heating, the feed temperature of the distillation column increased significantly to the saturated temperature of the extract stream. Thus, the reboiler duty of recovery column (DC-1) was drastically reduced. Moreover, the required condenser duty to cool the recycle solvent could also be decreased significantly. To further reduce the required condenser duty, the raffinate stream from the extractor could be used as the cold source since it has a low temperature (35 °C).

The boiling point of oleyl alcohol at 1 atm is 348 °C, which produces bottom stream at approximately that temperature. Instead of using the heat exchanger as a reboiler, it is necessary to utilize a furnace in the bottom sections of DC-1 and DC-2. Therefore, to minimize the significant problem due to the decomposition of 2,3-BDO at very high temperature, vacuum distillation was considered in the HED configuration. Similar to the conventional distillation configuration, restriction on using a heat exchanger as reboiler in this work are aimed at avoiding the potential for coking and fouling in the furnace. Besides, lower reboiler duty of the distillation column was also attained by lowering of the components’ boiling point under vacuum. Therefore, the pressures of DC-1 and DC-2 were designed to be the minimum pressure for the minimum allowable temperature in the condenser to utilize cooling water as the cooling medium (≥ 39 °C) and the maximum allowable bottom temperature to utilize high-pressure steam as the heating medium (244 °C).

**Fig. 10:**
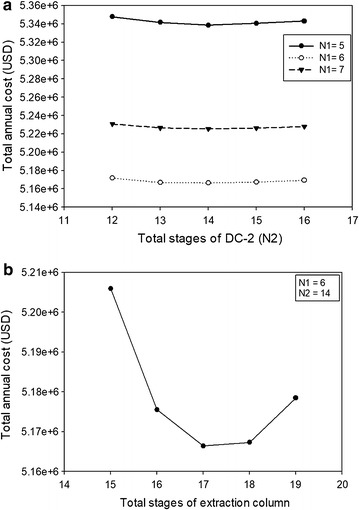
Summary of TAC plots for the hybrid extraction distillation: **a** at fixed number of extraction stages = 17, and **b** at fixed number of subsequent distillation column stages

**Fig. 11:**
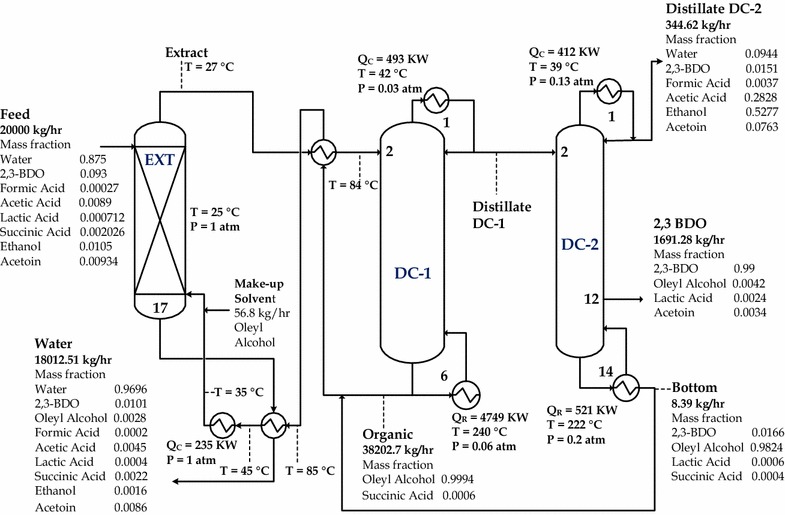
Optimal design of hybrid extraction distillation for the purification of 2,3-BDO

Based on the sequential iterative optimization procedure shown earlier (see Fig. 3), Fig. 10a summarizes the TAC plots at fixed number of extraction stages (17). Similar to the conventional distillation configuration, the optimal feed and product stages of DC-1 and DC-2 were also found while satisfying the internal hydraulic flow inside a distillation column. The optimal total stages for DC-1 and DC-2 were 6 and 14, respectively. By summarizing the minimized TAC at each number of distillation stages (shown in Fig. 10b), the optimal extraction stages was found to be 17, which required *S*/*F* = 1.91 to recover high purity 2,3 BDO in the side stream of DC-2. This *S*/*F* ratio can be derived from the minimum solvent necessary to achieve the product specification in the product stream with the lowest TAC. The optimal design for the proposed HED is shown in Fig. 11. It is found that significant reduction (54.8%) was obtained in reboiler duty, compared to the conventional distillation configuration. Much less water needs to be vaporized in the distillation column since most water is withdrawn from the extraction column as the raffinate stream.

#### Techno-economic analysis of proposed process configuration

**Fig. 12:**
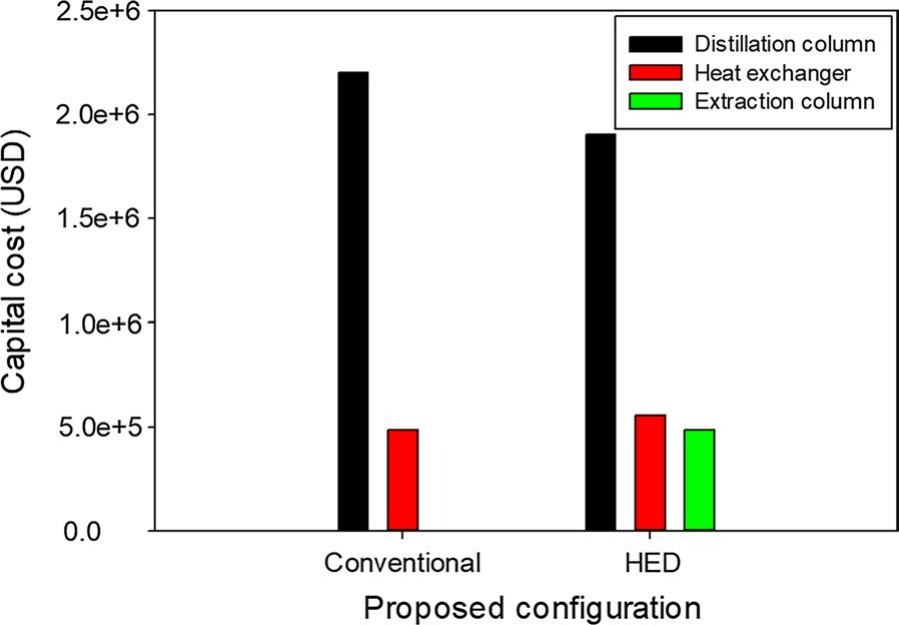
Comparison of capital costs for different process configurations

From the designs described, the proposed HED configuration requires more columns than the conventional distillation configuration. In addition, a heat exchanger is mandatory to re-cool the recycle solvent back to the extraction column, particularly when the utilization of product streams as heat sources of feed pre-heat were considered for both proposed configurations. Therefore, further discussions and conclusions require comparison of the performance of each configuration in terms of total capital cost, operating cost, and TAC. The detailed formula to evaluate the total capital cost, operating cost, and TAC can be found in the supplementary materials. Figure 12 compares capital costs of the different process configurations studied. Although the HED configuration requires a larger diameter of distillation column (particularly for DC-1) due to the larger amount of solvent, the required capital costs of two distillation columns in HED is lower than of the conventional single distillation configuration. It was observed that the capital cost of the heat exchanger required in reboiler and condenser duty of the distillation column is much higher than in the HED configuration. Although lower capital cost and lower reboiler duty of distillation column were attained using the HED configuration, it required higher capital cost (9.5% higher) than conventional distillation due to the requirement of the extraction column and heat exchangers.

**Fig. 13:**
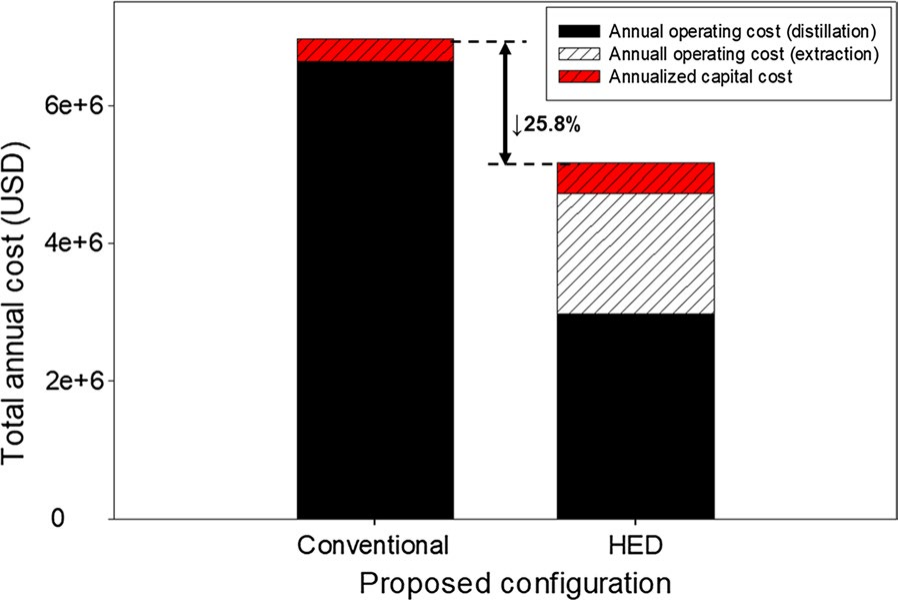
Comparison of TACs for different process configurations

Figure 13 compares the TACs of the two proposed configurations. Although HED has higher capital cost, the significantly lower reboiler and condenser duty in the distillation column result in 25.8% TAC saving relative to the conventional distillation process. Thus, it can be concluded that HED using oleyl alcohol solvent can be applied to the commercial purification of 2,3-BDO from its fermentation broth. However, in comparison with the reduction of reboiler duty that reached 54.8% (seen from Fig. 13), low TAC saving of HED is due to the high solvent operating cost. The high operating cost of solvent is due to the high price of oleyl alcohol that should be made-up during operation to compensate for the loss of oleyl alcohol. The raffinate from the extractor which will be sent back to the fermentation unit exhibited an excessive amount of solvent loss due to the solubility of solvent in water. Nevertheless, once the design considered that the feed stream from fermentation broth contains oleyl alcohol, greater TAC saving could be obtained since the solvent is sent back to the purification unit (HED).

## Conclusions

2,3-BDO capability towards many applications focuses attention on its production from biomass through the fermentation process. Fermenter output needs further processing to separate it from water and other soluble and insoluble contents. Several processes have been demonstrated to recover 2,3-BDO from fermentation broth but they still face challenges for commercial scale up. Therefore, this work proposes several process configurations to develop a cost-effective process for 2,3-BDO purification. To generate the proper thermodynamic models for the proposed design, binary parameters of the NRTL and UNIQUAC models for the main component and solvent mixture were obtained through rigorous regression and validation. The systematic solvent selection has been successfully implemented to determine a good solvent for the proposed HED configuration, and oleyl alcohol was selected for extraction of 2,3-BDO. The HED configuration successfully appeared as a promising technique when compared with the conventional distillation process since it reduced the required energy and TAC of the process. The proposed HED showed promise, particularly by including the benefits of using the heat exchangers for feed preheating and solvent cooling to obtain lower reboiler duty of the solvent recovery column and lower condenser duty for the recycle solvent. As a result, the proposed HED configuration drastically reduced the reboiler duty consumption and TAC by up to 54.8 and 25.8%, respectively.

## Additional file

**Additional file 1.** Thermodynamic Equations and Calculation Method for Techno-Economic Analysis.

## Abbreviations

2,3-BDO: 2,3-butanediol
HED: hybrid extraction-distillation
NRTL: non-random two liquid
UNIQUAC: universal quasi chemical
VLE: vapor liquid equilibrium
LLE: liquid liquid equilibrium
AAD: average absolute deviation
UNIFAC: universal quasichemical functional-group activity coefficients
DC: conventional column
EXT: extraction column
DC-1: recovery column
DC-2: purification column
SQP: sequential quadratic programming
TAC: total annual cost
*K*_*D*_: distribution coefficient
*α*: selectivity
OA: oleyl alcohol
*S*/*F*: ratio of solvent to feed flow rate
