# Supplementary material for: Purification of 2,3-butanediol from fermentation broth: process development and techno-economic analysis
Source: Biotechnol Biofuels. 2018 Jan 25;11:18. doi: 10.1186/s13068-018-1013-3 (PMC5785907; doi:10.1186/s13068-018-1013-3)
Supplement: Supplementary file 1 — Additional file 1. Thermodynamic Equations and Calculation Method for Techno-Economic Analysis. [file 13068_2018_1013_MOESM1_ESM.pdf]

Additional Material

"Thermodynamic Equations and Calculation Method for Techno-economic Analysis"

Purification of 2,3-Butanediol from Fermentation Broth: Process Development and Techno-economic Analysis

Gregorius Rionugroho Harvianto[1#], Junaid Haider[1#], Jimin Hong[1], Nguyen Van Duc Long[1], Jae-Jin Shim[1], Moo Hwan Cho[1], Woo Kyoung Kim[1], Moonyong Lee[1*]

[1]School of Chemical Engineering, Yeungnam University, Gyeongsan 712-749, Republic of Korea

Submitted to Biotechnology for Biofuels

*****Correspondence concerning this article should be addressed to:

Prof. Moonyong Lee

Address: School of Chemical Engineering, Yeungnam University, Dae-dong 214-1, Gyeongsan 38541, Republic of Korea

Email: mynlee@yu.ac.kr

Telephone: +82 53 810 3241

A. NRTL and UNIQUAC equations

1. NRTL equation:

$$\ln \gamma_i = \frac{\sum_j x_j \tau_{ji} G_{ji}}{\sum_k x_k G_{ki}} + \sum_j \frac{x_j G_{ij}}{x_k G_{kj}} \left(\tau_{ij} - \frac{\sum_m x_m \tau_{mj} G_{mj}}{\sum_k x_k G_{kj}} \right) \qquad (A.1)$$

$$G_{ij} = \exp(-\alpha_{ij} \tau_{ij}) \qquad (A.2)$$

$$\tau_{ij} = a_{ij} + \frac{b_{ij}}{T} \qquad (A.3)$$

$$\alpha_{ij} = c_{ij} + d_{ij}\mathrm{T} \qquad (A.4)$$

$$\tau_{ii} = 0 \qquad (\mathrm{A}.5)$$

$$G_{ii} = 1 \qquad (\mathrm{A}.6)$$

γ_i is the activity coefficient of molecule i, x is the mole fraction, τ_{ij} ($\neq \tau_{ji}$) is the interaction parameter between groups i and j, G_{ij} is as defined in equation 2, m is the measured data, k is Boltzmann's constant, α_{ij} is the non-randomness constant for binary ij interactions. a_{ij}, b_{ij}, and c_{ij} are binary interactions of $a, b, and\ c$ between a pair of groups i and j, respectively.

2. UNIQUAC equation:

$$\ln \gamma_i = \frac{\ln \frac{\Phi_i}{x_i} + \frac{z}{2} q_i \ln \frac{\Phi_i}{\theta_i} - q_i' \ln t_i' - q_i' \sum_j \theta_j' \tau_{ij}}{t_j' + \ l_i + q_i' - \frac{\Phi_i}{x_i} \sum_j x_j \, l_j} \qquad (A.7)$$

$$\theta_i = \frac{q_i x_i}{q_r};\ q_r = \sum_k q_k x_k \qquad (A.8)$$

$$\theta'_i = \frac{q'_i x_i}{q'_r};\ q'_r = \sum_k q'_k x_k \qquad (A.9)$$

$$\Phi_i = \frac{r_i x_i}{r_r};\ r_r = \sum_k r_k x_k \qquad (A.10)$$

$$l_i = \frac{z}{2}(r_i - q_i) + 1 - r_i \qquad (A.11)$$

$$t'_i = \sum_k \theta'_k \tau_{ki} \qquad (A.12)$$

$$\tau_{ij} = exp\left(a_{ij} + \frac{b_{ij}}{T} + c_{ij} \ln T\right) \qquad (A.13)$$

Φ is the segment fraction, θ is the area fraction, r and q are respectively pure component relative volume and surface area parameters, τ_{ij} ($\neq \tau_{ji}$) is the interaction parameter, and z is the lattice coordination number (equal to 10). The r and q values are obtained from the Aspen Plus databank.

B. Economic Evaluation of Proposed Configurations

1. Sizing the distillation column

A distillation column was determined to have a sieve tray with 0.61 m tray spacing. The column diameter was determined by the column flooding condition that fixes the upper limit of the vapor velocity. The operating velocity is usually between 70 and 90% of the flooding velocity [1, 2]; in this study, 80% of the flooding

velocity was used as the default. Column analysis in Aspen Plus®V9 was used to calculate the diameter of the column and ensure the satisfaction of hydraulic flow inside of the column.

2. Sizing the extraction column

Due to the number of stages required for the extraction column, an agitated extraction column was used in this work. The column diameter and height were determined from the methods provided by Todd [3]. The diameter of the column will be evaluated so that the column operates at 75% of the flood point.

3. Capital cost (CC)

Guthrie's modular method was applied [4]. In this study, the Chemical Engineering Plant Cost Index (CEPCI) 576.1 (2014) was used for cost updating.

CC = BMC column + BMC wall + BMC tray stack + BMC condenser + BMC reboiler (B. 1)

Updated bare module cost (BMC) = UF × BC × (MPF + MF − 1) (B. 2)

where UF is the update factor: $UF = \frac{present\ cost\ index}{bare\ cost\ index}$ (B. 3)

BC is the bare cost of the heat exchanger: $BC = BC_0\ x\ \left(\frac{A}{A_0}\right)^{\alpha}$ (B. 4)

where MPF is the material and pressure factor; MF is the module factor (a typical value), which is affected by the base cost.

Area of the heat exchanger, $A = \frac{Q}{U\ \Delta T}$ (B. 5)

Material and pressure factor: MPF = $F_m + F_p + F_d$ (B. 6)

where F_m, F_p, and F_d are the construction material, pressure variation, and design variation factors, respectively. Particularly for the HED configuration, the vacuum pump cost (C_{VP}) was calculated according to Ji et al. [5] and is presented in the following equations.

$$C_{VP} = 4200 \frac{CEPCI\ 2014}{CEPCI\ 1994}\left(\frac{60 g_T R T_0}{3600 P_0}\right)^{0.55} \quad (B.7)$$

where g_T is the total feed flow (kmol/h), R is the gas constant (0.0831 m^3 bar/kmol K), T_0 is the temperature at the standard condition (273.15 K), and P_0 is the pressure at the standard condition (1.013 bar).

4. Operating cost (OC):

Table A.1. Utilities cost data [6].

Utility	Price ($/GJ)
Cooling Water	0.35
Steam (High Pressure)	17.7
Electricity	16.80

$$OC = C_{HPS} + C_{CW} + C_E + C_{NG} \quad (B.8)$$

where C_{HPS} is the cost of the high-pressure steam; C_{CW} is the cost of cooling water; and C_E is the cost of electricity.

The power consumption of the vacuum pump (W_{VP}) and the condenser in the HED configuration were calculated according to Ji et al. [5] using the following equation, which involves the total feed flow. Because the distillate stream is condensed before the vacuum pump, the power consumption (W_{VP}) is over-predicted (but the influence of this on the total cost is small).

$$W_{VP} = 10\% \frac{g_T}{3600} R T_{intake} \frac{kr}{kr-1}\left[\left(\frac{P_{DIS}}{P_{OP}}\right)^{\left(\frac{kr-1}{kr}\right)} - 1\right] \quad (B.9)$$

where W_{VP} is in kW, g_T is the total feed flow (kmol/h), T_{intake} is the absolute temperature of vapor at the intake conditions (298 K), kr is the heat capacity ratio (1.33), P_{DIS} is the discharge pressure (1.013 bar), and P_{OP} is the condenser operating pressure in bar.

In addition, the price of oleyl alcohol (solvent) was 3,517 USD / ton, obtained from the MOLBASE database [7].

5. Total annual cost (TAC):

The total annual cost includes the annual capital cost (ACC) and the annual operating cost (AOC). The annual investment cost was obtained from the literature and refers to the annual payments over the life of the project [8].

$$ACC = CC\left[{i(1+i)^n}\Big/{(1+i)^n - 1} \right] \quad \text{(B.10)}$$

where *i* is the interest rate per year (8%), and *n* is the project duration (10 years).

$$TAC = ACC + AOC \quad \text{(B. 11)}$$

References

1. Premkumar R, Rangaiah GP: **Retrofitting conventional column systems to dividing-Wall Columns**. *Chemical Engineering Research and Design* 2009, **87**(1):47-60.
2. Sinnott RK: **Chemical Engineering Design** vol. 6, 4th edn. Oxford: Elsevier Butterworth Heinemann; 2005.
3. Todd DB: **8 - Solvent Extraction A2 - Vogel, Henry C**. In: *Fermentation and Biochemical Engineering Handbook (Second Edition).* Edited by Todaro CL. Westwood, NJ: William Andrew Publishing; 1996: 348-381.

4. Biegler LT, Grossmann, I.E. & Westerberg, A.W. : **Systematic methods of chemical process design**. New Jersey: Prentice Hall Inc.; 1997.

5. Ji W, Hilaly A, Sikdar SK, Hwang S-T: **Optimization of multicomponent pervaporation for removal of volatile organic compounds from water**. *Journal of Membrane Science* 1994, **97**(0):109-125.

6. R. Turton RCB, W.B. Whiting, J.A. Shaeiwitz, D. Bhattacharyya: **Analysis, synthesis and design of chemical processes**, 4th edn. Upper Saddle River, NJ: Prentice Hall; 2012.

7. **CAS No. 143-28-2 Oleyl alcohol** [http://www.molbase.com/en/cas-143-28-2-cate-p1n1-p6n4011-p7n10-p4n2.html]

8. Hicks T, Chopey N: **Handbook of Chemical Engineering Calculations, Fourth Edition**: McGraw Hill Professional; 2012.
